# The age-associated loss of ischemic preconditioning in the kidney is accompanied by mitochondrial dysfunction, increased protein acetylation and decreased autophagy

**DOI:** 10.1038/srep44430

**Published:** 2017-03-15

**Authors:** Stanislovas S. Jankauskas, Irina B. Pevzner, Nadezda V. Andrianova, Ljubava D. Zorova, Vasily A. Popkov, Denis N. Silachev, Nataliya G. Kolosova, Egor Y. Plotnikov, Dmitry B. Zorov

**Affiliations:** 1 Belozersky Institute of Physico-Chemical Biology, Lomonosov Moscow State University, 119992, Leninskye Gory, House 1, Building 40, Moscow, Russia; 2 International Laser Center, Lomonosov Moscow State University, 119992, Leninskye Gory, House 1, Building 62, Moscow, Russia; 3 Faculty of Bioengineering and Bioinformatics, Lomonosov Moscow State University, 119992, Leninskye Gory, House 1, Building 73, Moscow, Russia; 4 Institute of Cytology and Genetics, Novosibirsk, Russia

## Abstract

In young rats, ischemic preconditioning (IPC), which consists of 4 cycles of ischemia and reperfusion alleviated kidney injury caused by 40-min ischemia. However,old rats lost their ability to protect the ischemic kidney by IPC. A similar aged phenotype was demonstrated in 6-month-old OXYS rats having signs of premature aging. In the kidney of old and OXYS rats, the levels of acetylated nuclear proteins were higher than in young rats, however, unlike in young rats, acetylation levels in old and OXYS rats were further increased after IPC. In contrast to Wistar rats, age-matched OXYS demonstrated no increase in lysosome abundance and LC3 content in the kidney after ischemia/reperfusion. The kidney LC3 levels were also lower in OXYS, even under basal conditions, and mitochondrial PINK1 and ubiquitin levels were higher, suggesting impaired mitophagy. The kidney mitochondria from old rats contained a population with diminished membrane potential and this fraction was expanded by IPC. Apparently, oxidative changes with aging result in the appearance of malfunctioning renal mitochondria due to a low efficiency of autophagy. Elevated protein acetylation might be a hallmark of aging which is associated with a decreased autophagy, accumulation of dysfunctional mitochondria, and loss of protection against ischemia by IPC.

Aging affects both the kidney’s morphology and functioning and it is associated with the increased sensitivity of the kidney to the damaging factors leading to a more severe course of acute kidney injury (AKI) and increased risk of transition from AKI to chronic kidney disease.

Among the molecular mechanisms of aging, an important role is assigned to the processes of acetylation^1^,^2^. It is assumed to be a component of the beneficial mechanism of caloric restriction which can increase lifespan through the system associated with the expression of Sirt deacetylases^3^,^4^,^5^. Another target of caloric restriction is mTOR, which controls the processes of autophagy^6^,^7^. The decrease in autophagy is an important attribute of the aging cell^8^. Finally, mitochondria have been implicated in a number of processes, that are crucial in aging^9^,^10^,^11^,^12^. Distinct functional and morphological changes appear to accumulate in the mitochondria of the aged organ^11^,^12^. Currently, these three elements: mitochondria, levels of protein acetylation and autophagy have become critical targets in studies of protection against age-related deleterious changes in organs such as the kidney.

Aging is accompanied by ischemia-associated pathologies and important protective anti-ischemic measures are ischemic preconditioning (IPC) and post conditioning^13^,^14^,^15^ including both direct and remote^16^, shown for a large number of organs and tissues^17^,^18^,^19^,^20^, including the kidney^21^,^22^. The studies of signaling pathways involved in IPC^21^,^23^,^24^ showed that mitochondria are an essential requisite for implementation of protective mechanisms and dysfunction of these organelles may be related with age-dependent changes in the efficiency of IPC^25^.

In a contradictory manner, while the majority of patients with AKI are elderly, the plethora of experimental data on this pathology was obtained on young animals. This may be an explanation why most clinical trials of drugs fail although their efficacy has been proven in animal studies. At the same time, the use of old animals is coupled with objective difficulties, and therefore, an important challenge is to find models that examine the phenomenon of aging *in vivo*. One such model is accelerated-senescence OXYS rats. This strain was established by selection and inbreeding of Wistar rats sensitive to the cataractogenic effects of a galactose-rich diet. Thereafter, in rats of this strain, cataract, a typical disease of old age, developed spontaneously at an early age. OXYS rats demonstrate the signs of premature aging manifested in the development of diseases common to elder age, such as early retinopathy similar to age-related macular degeneration in humans, osteoporosis, arterial hypertension, hypertrophic cardiomyopathy, accelerated thymus involution, sarcopenia and accelerated brain senescence with the features inherent for Alzheimer’s disease^26^,^27^,^28^,^29^,^30^,^31^. It can be assumed that the accelerated aging also affects kidney, making these rats a unique model for studying the effects of ageing on renal diseases.

Presently it is unknown whether the protective effect of IPC is sustained in the kidney during advanced ageing. The goal of this study is to analyze possible changes in natural ischemic tolerance mechanisms in the aged kidney with special attention to protein acetylation, lysosomes and components of mitochondrial autophagy.

## Results

### Renal hemodynamics after ischemia and IPC

**Figure 1:**
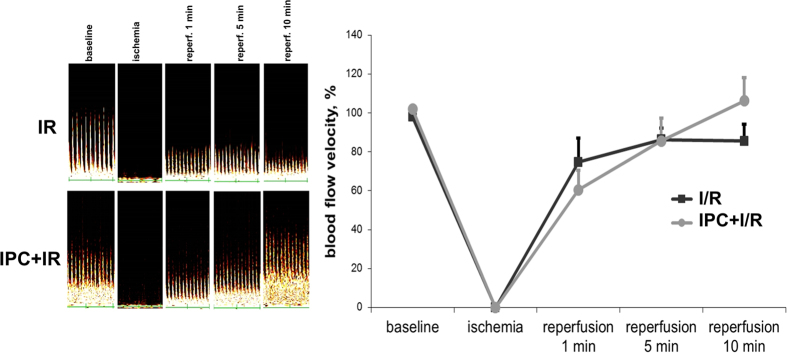
Renal hemodynamics after ischemia and IPC. Left, real-time tracings of the intrarenal arterial blood flow. Changes in response to I/R are presented. Right, mean blood flow velocity in the kidney of rats exposed to I/R with and without IPC. For “I/R young” group n = 6 and for “IPC + I/R young” group n = 5.

To study the alterations of renal vasculature in response to ischemia/reperfusion (I/R) we measured intrarenal blood flow velocity with Doppler ultrasound. With I/R, the renal blood flow in young rats reached only 80% of the pre-ischemic level after 10 min of reperfusion (Fig. 1). IPC (4 cycles of 15 seconds of ischemia and 15 seconds of reperfusion each) prior to I/R had a beneficial effect on blood flow restoration (Fig. 1).

### IPC affords protection of only young animals’ ischemic kidney

**Figure 2:**
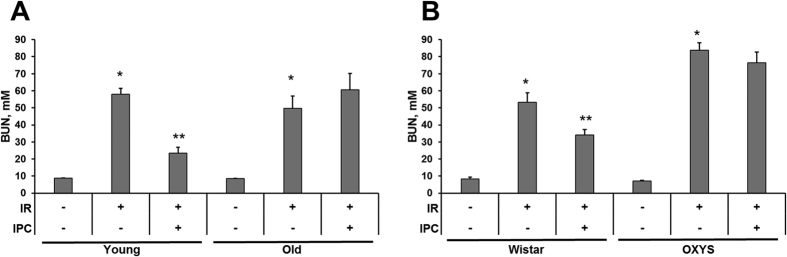
Acute kidney injury, as measured by blood urea nitrogen (BUN) levels in control rats, 48 hours after I/R and after I/R with IPC. (**A**) Young (4–6 month) and old (20–23 month) outbred rats. Number of animals: “young control” n = 11, “young I/R” n = 21, “young IPC+I/R” n = 10, “old control” n = 6, “old I/R” n = 6, “old IPC+I/R” n = 9. (**B**) Young (6-month) Wistar and OXYS rats. Number of animals: “Wistar control” n = 3, “Wistar I/R” n = 8, “Wistar IPC+I/R” n = 6, “OXYS control” n = 3, “OXYS I/R” n = 7, “OXYS IPC+I/R” n = 7. *p < 0.05 compared to control, **p < 0.05 compared to I/R.

Kidney I/R resulted in the development of AKI in young (4–6-month-old) rats (Fig. 2A). The impairment of excretory renal function was evidenced by more than 6-fold increase in blood urea (BUN) 48 hours after I/R. IPC prior to I/R alleviated the severity of renal failure in young animals (Fig. 2A). Although the severity of renal failure after I/R in 20–23-month-old rats was about the same as in young rats, IPC of the kidneys of old animals was not protecting (Fig. 2A). Of note, BUN of intact 20–23-month-old rats was the same as in young rats (Fig. 2A).

In young rats, the total antioxidant capacity (TAC) of cytosolic fractions of the kidney exposed to IPC was increased by 33% versus that in unexposed animals (from 45.82 ± 0.75 to 61.14 ± 3.66 mmol of trolox-equivalents/mg protein). Unlike in young rats, in old ones TAC decreased after IPC by 43% versus unexposed old rats (from 57.91 ± 14.66 to 33.00 ± 8.20).

### The effect of IPC on AKI in OXYS

We have not found the signs of impairment of renal function in 6-month-old OXYS. No significant differences in renal morphology in OXYS rats compared to Wistar were found (Supplementary Figure S1). Kidney I/R in these rats resulted in a profound renal insufficiency with 12-times BUN increase (Fig. 2B). In addition, IPC of 6-month-old OXYS didn’t afford nephroprotection against I/R (Fig. 2B). This response to IPC was quite different from the effect of IPC in young, but similar to the response of old outbred rats, which also demonstrated insensitivity to renal IPC (Fig. 2A).

To exclude the possibility that the response to IPC is due to the difference in the strains of the rats, we studied the kidney functioning in 6-month Wistar rats (which were the origin for raising OXYS) 48 hours after I/R without and with IPC (Fig. 2B). In these age-matched Wistar, BUN was normal and close to that in outbred animals. On the day 2 after I/R, BUN in these animals was increased to 6 times versus control level. IPC of 6-month Wistar as in young outbred rats had a remarkable nephroprotective effect, resulting in reducing BUN (Fig. 2B). Thus, we found no differences in the occurrence of ischemia-induced AKI in young outbred and Wistar rats.

In addition, in intact OXYS, renal TAC was slightly higher than in age-matched Wistar rats (54.68 ± 10.16 and 43.31 ± 4.23, respectively) and after IPC, it decreased in OXYS kidneys to 28.10 ± 2.18 mmoles of trolox-equivalents/mg protein.

### Proteins acetylation in young and old animals

**Figure 3:**
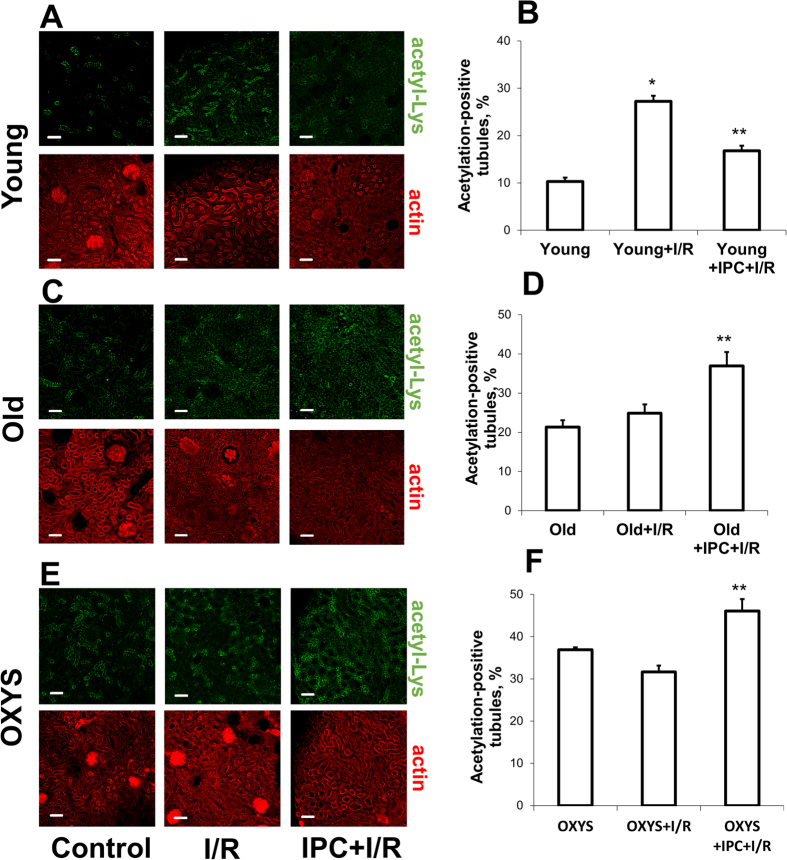
Protein acetylation in renal cortex. Kidneys of young (**A,B**), old (**C,D**) and young OXYS (**E,F**) rats were harvested at baseline, 40 minutes after I/R and I/R with IPC. Graphs in (**B,D,F**) illustrate the portion of tubules with acetylated nuclei. (**A,C,E**): Confocal microscopy of kidney cortex sections stained with acetyl-Lys antibodies (green fluorescence) and TRITC-phalloidin (red fluorescence). Scale bars indicate 100 μm. Number of slices: “young control” n = 45, “young I/R” n = 45, “young IPC+I/R” n = 45, “old control” n = 60, “old I/R” n = 60, “old IPC+I/R” n = 45, “OXYS control” n = 45, “OXYS I/R” n = 60, “OXYS IPC+I/R” n = 60. *p < 0.05 compared to control, **p < 0.05 compared to I/R.

We found that in renal cortex, acetylated lysines are predominantly localized in the nuclei of tubular cells (Fig. 3A). Only 10% of the tubules from young rats have over half of nucleus with apparent fluorescent signal. Such acetylation-positive tubules were twice as abundant in kidneys harvested 40 minutes after I/R compared to baseline (Fig. 3A,B). If before I/R the kidney was subjected to IPC, the levels of acetylated nuclear proteins were significantly lower that after I/R alone (Fig. 3A,B).

The levels of acetylation in the old kidney cortex revealed a significant difference from those of young ones (Fig. 3C). The percentage of acetylation-positive tubules was two times more than in young animals (Fig. 3D vs 3B). I/R of the old kidney led only to a slightly higher number of acetylated tubules (Fig. 3C,D) while priming the kidney with IPC resulted in a remarkably greater number of these (Fig. 3C,D).

The response of kidney cortex acetylation levels to I/R and IPC in 6-month-old OXYS was qualitatively similar to that observed in 23-month-old outbred rats (Fig. 3E). In the kidneys of intact OXYS rats, the percentage of highly acetylated tubules was much higher than in the kidneys of young outbred rats (Fig. 3F) and was not higher in kidneys subjected to I/R. However, after IPC, a significantly greater percentage of highly acetylated tubules was observed (Fig. 3E,F).

### Response of autophagy-lysosomal machinery to I/R in young and OXYS rats’ kidneys

**Figure 4:**
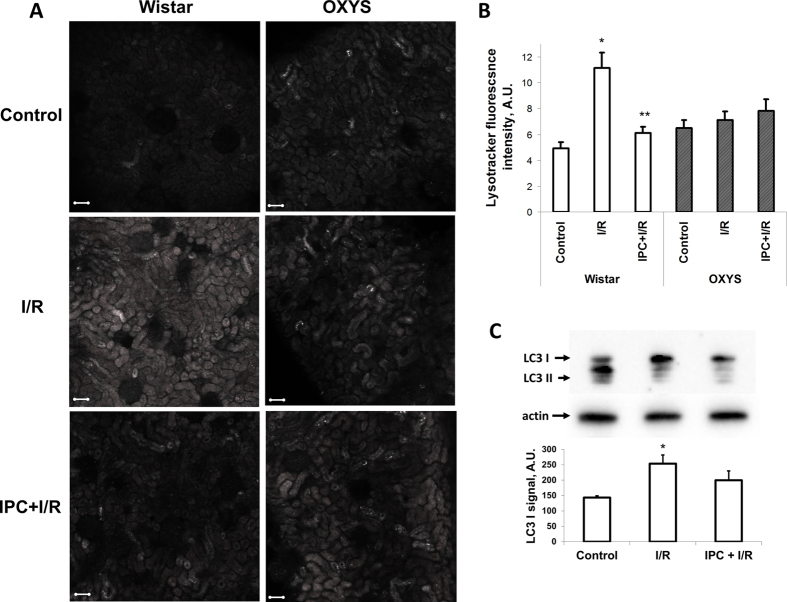
Autophagic and lysosomal activity in kidney after I/R. (**A**) Confocal microscopy after Lysotracker Green staining of vital renal cortex slices. The scale bar indicates 100 μm. (**B**) Quantification of Lysotracker Green fluorescence intensity. Number of slices: “Wistar control” n = 75, “Wistar I/R” n = 75, “Wistar IPC+I/R” n = 75, “OXYS control” n = 45, “OXYS I/R” n = 45, “OXYS IPC+I/R” n = 30. (**C**) Level of autophagy-associated LC3-I protein in kidneys harvested at baseline, 48 hours after I/R and after I/R with IPC, as estimated by Western blotting. Number of animals: “Wistar control” n = 3, “Wistar I/R” n = 4, “Wistar IPC+I/R” n = 3 *p < 0.05 compared to control, **p < 0.05 compared to I/R.

Differences between OXYS and young outbred rats were also evident in the response of autophagy-lysosomal machinery to kidney I/R. In renal cortex slices from intact Wistar rats, we observed uniform Lysotracker staining in the tubules (Fig. 4A). In kidneys harvested 24 hours after I/R, the average intensity of Lysotracker fluorescence in the slices of renal cortex was about twofold higher than in intact rat (Fig. 4A,B). In renal cortex slices from rats exposed to renal IPC before I/R, the average Lysotracker fluorescence intensity was similar to that in intact rats (Fig. 4A,B). The lysosomal staining of renal cortex slices from intact OXYS rats revealed a similar average Lysotracker fluorescence intensity compared to intact Wistars (Fig. 4A,B). However, unlike in age-matched Wistar rats, lysosome abundance 24 hours after I/R or after I/R with IPC was not greater than in controls (Fig. 4A,B).

To verify that the changes in lysosome abundance after I/R are due mostly to autophagy-lysosomal machinery, we evaluated the content of the autophagosomal marker LC3 in kidney homogenates of Wistar rats. The response of LC3-I content to I/R and IPC-I/R was similar to that observed in Lysotracker staining (Fig. 4C).

### Impaired autophagy and mitophagy in old rats’ kidneys

**Figure 5:**
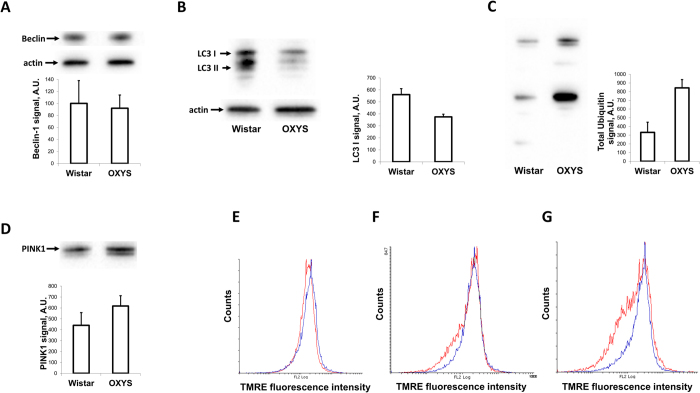
(**A**) Level of Beclin-1 in total kidney homogenates from Wistar and OXYS rats (**B**). Levels of LC3-I protein in total kidney homogenates from Wistar and OXYS rats. (**C**) Level of ubiqitinated proteins in isolated kidney mitochondria from Wistar and OXYS rats (**D**) Levels of PINK-1 in isolated kidney mitochondria of Wistar and OXYS rats. Number of animals: “Wistar” n = 3, “OXYS” n = 3. (**E**–**G**) Mitochondrial transmembrane potential in kidney mitochondria. Flow cytometry of isolated kidney mitochondria loaded with TMRE. (**E**) Young rats’ mitochondria in control (blue histogram) and 60 minutes after IPC (red histogram). (**F**) Transmembrane potential in kidney mitochondria from intact young (blue histogram) and old (red histogram) rats. A fraction of mitochondria with low transmembrane potential is seen as a left shoulder on each histogram. (**G**) Old rats’ mitochondria in control (blue histogram) and 60 minutes after IPC (red histogram). Note the increased fraction of de-energized mitochondria in old rats after IPC versus control rats. Number of animals n = 3 in all groups.

A study of the basal level of autophagy in the kidney revealed the same levels of Beclin-1 but considerably lower LC3-I content in intact OXYS rats compared to Wistar rats (Fig. 5A,B). This proves that already at the age of 6-months, OXYS show the decreased levels of autophagy that are typical for aged tissue^8^. In accordance with these data, the contents of ubiquitinated proteins and PTEN-induced putative kinase1 (PINK1) in kidney mitochondria of OXYS rats were significantly higher (Fig. 5C,D) than in age-matched Wistar rats, suggesting impaired mitophagy in OXYS. Since intact mitochondria are shown to be essential for the function of protective mechanisms involved in IPC^13^,^14^,^15^ we studied the function of mitochondria isolated from kidneys primed and unprimed with IPC. IPC in young animals did not cause any noticeable effect in the functioning of mitochondria, as assessed by their membrane potential values (Fig. 5E). Mitochondria isolated from the kidney of old rats differed from mitochondria of young rats by exhibiting a remarkably higher fraction with low transmembrane potential (Fig. 5F) and this fraction was even higher with IPC (Fig. 5G).

## Discussion

The phenomenon of IPC was introduced in 1986 in a model of myocardial infarction^17^, where brief periods of I/R afforded protection of the tissue from damage caused by subsequent prolonged ischemia. Some elements of kidney IPC have been documented in 1990 and 1994^32^,^33^. Nowadays, to prevent I/R-induced renal failure, various protocols of IPC are used generally containing several (1–6) episodes of I/R, each lasting 4–11 min^34^,^35^,^36^,^37^,^38^.

In this work, we explored the effects of IPC afforded by brief (15 seconds) intermittent episodes of I/R on processes occurring in old and young kidney which are experiencing ischemia-induced AKI. Priming of the kidney prior to an extensive ischemia alleviated damaging effect of I/R on the organ as was evidenced by a drop of BUN.

High autophagic activity in cells is often associated with tissue damage. The levels of lysosomal abundance in the kidney exposed to I/R and the key marker of autophagy LC3-I in the ischemic kidney were significantly increased. Priming the kidney with IPC resulted in relative normalization of these indicators of autophagy activation.

The increase in LC3-I demonstrates the activation of autophagic signaling pathways. Potent inducers of this process are ROS^39^ and ischemia (possibly as a source of ROS^40^). A key role of mitochondria in the pathogenesis of ischemia-related pathologies^41^ and specifically in AKI^42^ is well recognized. I/R causes mitochondrial dysfunction provoking excessive production of ROS by mitochondrial enzymes^41^. ROS may promote induction of the permeability-transition-pore in mitochondria (mPTP)^43^ through the mechanism of the ROS-induced-ROS-release^44^ which serves as a deadly signal for apoptotic execution of the cell. It is accepted that the ultimate goal of protective signaling pathways in the IPC cascade is the prevention of mPTP-opening^42^,^45^. Apparently, alleviation of the ischemic kidney injury by IPC associated with lower autophagy is the evidence for normalization of mitochondrial functioning and accompanying ROS-production.

We have demonstrated that renal protection against ischemic injury may be reached even when the duration of ischemic episodes is reduced from the conventional few minutes to 15 seconds. Apparently, within such a short period of ischemia, a trigger activating the protective signaling cascade is strong enough to induce ischemic tolerance and, therefore, the use of longer (5–10 minutes) ischemic episodes for mPTP-inhibition seems redundant. The most convincing evidence for affording ischemic tolerance as a result of IPC is an increase in total antioxidant capacity of renal tissue. Indirect evidence lies in the fact that to reduce the severity of renal failure after I/R by postconditioning, a few cycles of I/R episodes lasting 10–30 sec^46^ can be used. However, the ability to protect the organ from I/R-induced injury by an ultra-short preconditioning protocol has never been used.

The findings of the efficiency of such an ultra-short IPC can have a practical value. Such short protocol obviously, has no direct damaging effect on the tissue, and makes it possible to use IPC just before surgery on the organ eliciting the high risk of developing AKI.

Although experimental IPC models have been shown to evoke potent anti-ischemic defence^47^, IPC-induced protection is diminished in old animals, affording no protection in ischemic heart, brain and liver^25^,^48^,^49^,^50^,^51^,^52^. Renal protection by IPC in aged animals has not been tested yet. In this study, we, for the first time, demonstrate that in old animals the protective effect of IPC on the renal function vanishes and the level of kidney failure in exposed aged unprimed animals to I/R is as high as in primed aged animals. Since the cardioprotective effect of IPC could be restored in old animals performing treadmill exercises with normalization of mitochondrial functioning, it gives an additional argument to consider mitochondria as a key player in lost preconditioning effects with aging^53^.

To get insight into a mechanism for the age-dependent loss of a protective mechanism of IPC, we tested a hypothesis that some deleterious alterations of proteins participating in protective pathways may occur. Acetylation, an important post-translational modification of proteins, possibly plays a key role in the mechanism of aging. Thus, the activation of the deacetylase Sir2 was shown to increase the lifespan of yeast^54^ and worms^55^ and the knockout of Sirt1 (a Sir2 homolog in mammals) results in a decrease in lifespan of mice and abolishes the positive effect of caloric restriction on lifespan^56^. Inhibition of acetyltransferase, catalyzing acetylation, increased the lifespan of yeast^57^ and Drosophila^58^, while the increase in cytosolic acetyl-CoA which acetylates proteins in a non-catalytical way^59^ accelerated cellular senescence^58^. These data indicate that a high level of proteome acetylation is probably the cause of those negative processes that are associated with aging. In this study, we confirmed that in renal tissue of old rats the level of nuclear protein acetylation is higher, and this phenomenon is associated with the loss of the protective effect of IPC.

The most common site for acetylation of proteins is the amino-group of lysine and it also is the main site of ubiquitination. Therefore, these two processes may be in a competitive interaction. It can make acetylation as an alternative to the mechanism of disposal of impaired proteins through ubiquitination. Indeed, acetylation prevented the proteasomal degradation of proteins^60^. Thus, excessive acetylation and low activity/expression of deacetylases in the cell resulting in increased levels of defective proteins would neutralize the efficiency of the cellular system of quality control. This may explain the loss of the protective action of the IPC in aged animals, as ubiquitination of proteins including mitochondrial plays a key role in segregation and destruction of dysfunctional cell components, including organelles. The mechanism of mitochondrial quality control is based on the principle of homeostasis of the mitochondrial transmembrane potential. When it drops as a result of impairment in oxidative phosphorylation (which could be also age-dependent), it cannot drive PINK1 into the mitochondria where it is normally cleaved by the presenilins-associated rhomboid-like protein (PARL). As a result, the PINK1 remains on the outer membrane of dysfunctional mitochondrion where it facilitates the ubiquitination by Parkin^61^ which is a starting point for disposal of impaired mitochondrion through autophagy/mitophagy.

Our data shows that in OXYS rats demonstrating signs of premature aging, the levels of protein ubiqutination and the content of PINK1 in the kidney mitochondria were elevated demonstrating an accumulation of impaired mitochondria. This suggests that although the OXYS kidney accumulates more low-functional mitochondria, the process of their elimination through mitophagy is retarded. Indeed, in the entire population of mitochondria isolated from the kidney of old rat, there was an essential portion of mitochondria with low membrane potential. While in young rats the IPC of the kidney does not cause changes in the membrane potential of their mitochondria, in old animals the IPC causes an increase of low-potential mitochondrial population, i.e., instead of IPC beneficial effect, we observed a deleterious effect of IPC in aged animals. These changes were associated with higher level of protein acetylation.

Thus, a high level of acetylated proteins in old animals is associated with accumulation in the kidney of partially de-energized mitochondria which are more susceptible to oxidative stress^41^. The level of protein acetylation in young OXYS was very high, even higher than in the 23-month-old outbred rats. IPC leads to an increased ischemic tolerance apparently by lowering the production of oxidants and increasing the antioxidative redox buffering capacity. In the renal tissue of young animals, excessive ROS are quickly scavenged by a redox buffer. As a result, the IPC-induced ROS-burst has enough time to ignite the protective signaling pathway but not enough to activate the damaging cascade. Our data that IPC improves the total antioxidative capacity support this suggestion. On the contrary, in old rats IPC causes an essential drop of ROS-buffering capacity. Under these conditions, an IPC-induced ROS-burst develops a profound oxidative stress which is high enough to damage tissue rather than activate protective signaling. Possibly, oxidative stress is either a cause or a result of enhanced levels of acetylation leading to limited healing ability due to a low autophagic activity in old animals.

## Materials and Methods

### Animals

Male outbred rats of age 4–6 and 20–23 months and 6-month-old Wistar and OXYS rats were used. The animal protocols were evaluated and approved by the animal ethics committee of Belozersky Institute of Physico-Chemical Biology (Protocol 2/13 from April 8, 2013). They were in accordance with the Federation of Laboratory Animal Science Associations (FELASA) guidelines.

### I/R and IPC protocols

The animals anesthetized with chloralhydrate (300 mg/kg, i.p.) were subjected to 40-minute warm ischemia of the left kidney as described^21^,^62^. The renal vascular bundle was occluded with a microvascular clip for 40 minutes. Circulation was restored by removing the clip. The lack of blood flow during ischemia (Fig. 1) and renal hemodynamics during reperfusion were assessed by 25-MHz ultrasound Doppler transducer (Doppler Minimax, Russia). Nephrectomy of the right kidney was executed simultaneously with ischemia of the left one. During operation, the body temperature of the rat was maintained at 37 ± 0.5 °C.

IPC was performed by clamping renal arteries with a microvascular clip and consisted of 4 cycles with 15-second ischemia and 15-second reperfusion each, immediately before the clamping of renal pedicle for 40 minutes. Blood samples were taken 48 hours after ischemia to determine BUN using AU480 Chemistry System (Beckman Coulter, USA).

### Immunofluorescence

Kidney slices were fixed for 24 hours in 4% formaldehyde with PBS at 4 °C, and permeabilized in 0.02% Triton X-100 for 60 minute at 20 °C. Sections were incubated overnight with 1:200 anti-acetyl-Lys Ab (CellSignaling, USA) and after three rinses in PBS were incubated 1 hour with secondary antibodies diluted 1:200 (FITC-conjugated anti-rabbit IgG; Jackson ImmunoResearch Laboratories, USA). For actin staining, kidney slices were incubated with 1:100 Phalloidin-TRITC (Invitrogen, USA).

### Lysosome abundance

Kidneys were excised 24 hours after I/R and placed in the incubation medium (Hank’s Balanced Salt Solution for cell cultures added with 10 mM Hepes-NaOH, pH 7.4) to wash out the blood. Then, 10–15 μm thick sections through the cortical zone of the kidney were made. Tissue sections were washed using the incubation medium (all procedures and incubation were done at 25 °C) and stained for 30 minutes with 1 μM Lysotracker Green (Invitrogen, USA).

### Confocal microscopy

Kidney slices after Lysotracker or immune staining were imaged with an LSM510 inverted confocal microscope (Carl Zeiss, Jena, Germany). Images were processed using ImageJ software (NIH, Bethesda, MD, USA).

### Western blotting

Kidney homogenate samples were loaded onto 15% Tris–glycine polyacrylamide gels (10 μg protein/lane). After electrophoresis, gels were blotted onto PVDF membranes (Amersham Pharmacia Biotech, UK). Membranes were blocked with 5% non-fat milk in PBS/0.1% Tween-20 and subsequently incubated with primary antibodies: anti-LC3 A/B (CellSignaling, USA), anti-Beclin-1 1:1000 (CellSignaling, USA), anti-ubiquitin - polyclonal rabbit 1:1000 (Abcam, USA), anti-B-actin monoclonal mouse 1:1500 (Sigma, USA), anti-PINK1 polyclonal rabbit 1:1000 (Abcam, USA). Membranes were stained with secondary antibodies: anti-mouse IgG or anti-rabbit IgG conjugated with horseradish peroxidase 1:10000 (Jackson ImmunoResearch, USA). Detection was performed by V3 Western Blot Imager (BioRad, USA).

### Mitochondria isolation

The rat kidney mitochondria were isolated by differential centrifugation in the medium containing 0.25 M sucrose, 10 mM Tris-HCl, 1 mM EDTA, 0.1% BSA, pH 7.4 and incubated in the same medium without BSA, EDTA, or DTT. Protein concentration was measured by bicinchoninic acid assay (Sigma, USA).

### Flow cytometry

Flow cytometry was performed by using a Cytomics FC500 (Beckman Coulter, USA). TMRE-mediated fluorescence reflecting a magnitude of transmembrane potential was measured on the FL2-channel with λ_ex_ = 488 nm. The incubation medium contained 120 mM KCl, 3 mM HEPES, 1 mM EGTA, 5 mM K_2_PO_4_, 100 nM TMRE, 5 mM succinate, and 200 μg mitochondrial protein/ml, pH 7.2–7.4.

### Total antioxidative capacity

TAC of kidney homogenates was measured by the hemoglobin-hydrogen peroxide-luminol chemiluminescence. Briefly, 10 μl of the sample were added to 100 μl of reaction medium (0.21 μM hemoglobin, 10 μM luminol in PBS) and the reaction was initiated by 20 μM H_2_O_2_. TAC was measured by assessing the luminescence quenching through scavenging the radicals generated by hemoglobin-hydrogen peroxide. The outcomes were expressed in mmoles of Trolox equivalent per mg protein.

### Statistics

All groups contained 6–10 animals. Western blots, immunohystochemistry, lysotracker staining and flow cytometry experiments were performed at least in triplicate. Data are presented as mean ± SEM. Comparisons between groups were made using a Student t-test with a P ≤ 0.05 taken to indicate statistical significance.

## Additional Information

**How to cite this article**: Jankauskas, S. S. *et al*. The age-associated loss of ischemic preconditioning in the kidney is accompanied by mitochondrial dysfunction, increased protein acetylation and decreased autophagy. *Sci. Rep.* **7**, 44430; doi: 10.1038/srep44430 (2017).

**Publisher's note:** Springer Nature remains neutral with regard to jurisdictional claims in published maps and institutional affiliations.

## Supplementary Material

Supplementary Figures
