# Supplementary material for: The age-associated loss of ischemic preconditioning in the kidney is accompanied by mitochondrial dysfunction, increased protein acetylation and decreased autophagy
Source: Sci Rep. 2017 Mar 15;7:44430. doi: 10.1038/srep44430 (PMC5353572; doi:10.1038/srep44430)

The age-associated loss of ischemic preconditioning in the kidney is accompanied by mitochondrial dysfunction, increased protein acetylation and decreased autophagy

Stanislovas S. Jankauskas[1], Irina B. Pevzner[1], Nadezda V. Andrianova[1], Ljubava D. Zorova[1,2], Vasily A. Popkov[3], Denis N. Silachev[1], Nataliya G. Kolosova[4], Egor Y. Plotnikov[1,*], Dmitry B. Zorov[1,*]

[1]Belozersky Institute of Physico-Chemical Biology, Lomonosov Moscow State University, 119992, Leninskye Gory, House 1, Building 40, Moscow, Russia

[2]International Laser Center, Lomonosov Moscow State University, 119992, Leninskye Gory, House 1, Building 62, Moscow, Russia

[3]Faculty of Bioengineering and Bioinformatics, Lomonosov Moscow State University, 119992, Leninskye Gory, House 1, Building 73, Moscow, Russia

[4] Institute of Cytology and Genetics, Novosibirsk, Russia

**Corresponding authors e-mail addresses: plotnikov@genebee.msu.ru, zorov@belozersky.msu.su*

Short title: Lack of ischemic preconditioning in aged kidney

Supplementary Figure S1 Renal histology of Wistar and OXYS rats. Images show kidney cortex (A) and medulla (B) sections stained with hematoxylin and eosin.

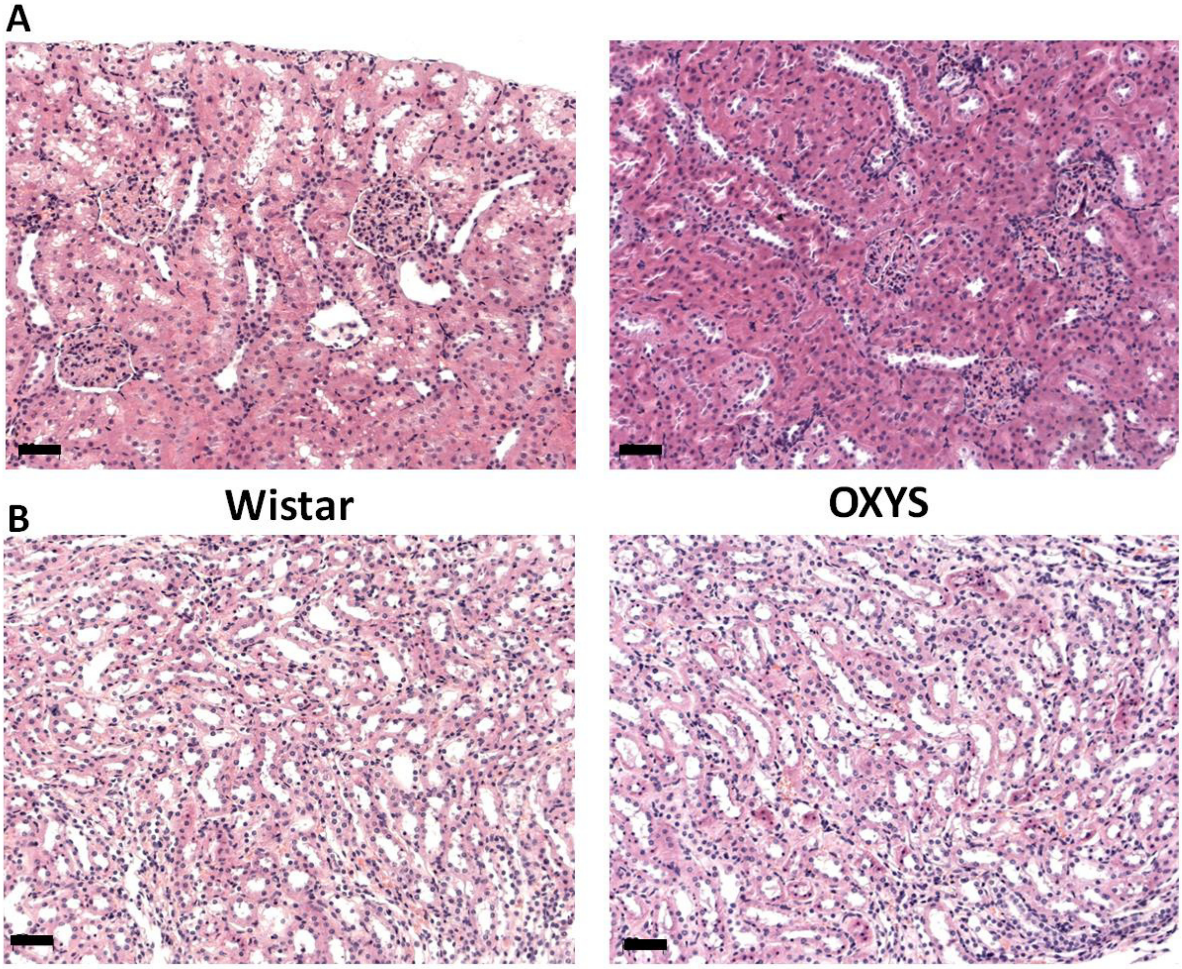

Supplementary Figure S2 Autophagic and lysosomal activity in kidney after I/R.
Figure S2 showed the raw uncropped images of figure 4C.

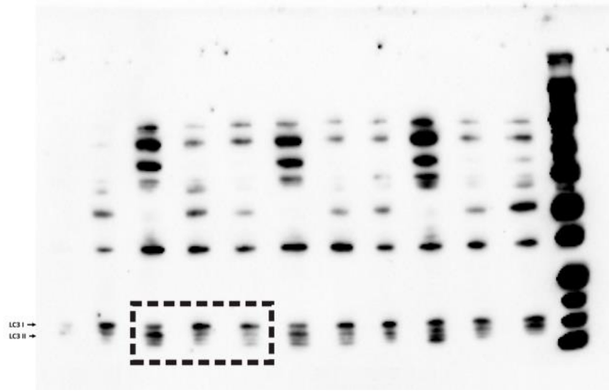

Supplementary Figure S3. Autophagy and mitophagy in kidney of OXYS or Wistar rats.
Figure S3 showed the raw uncropped images of figure 5A,B,C,D.

Figure 5A

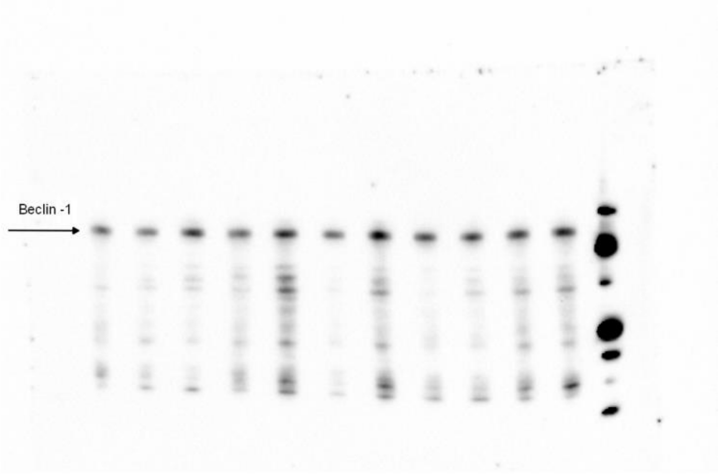

Figure 5B

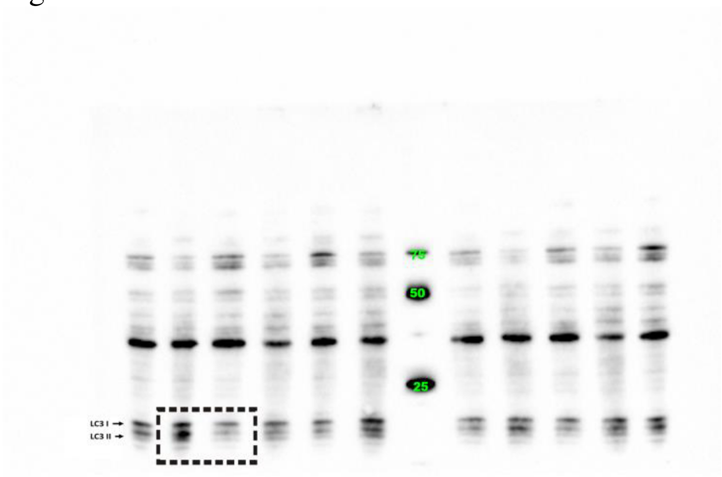

Figure 5C

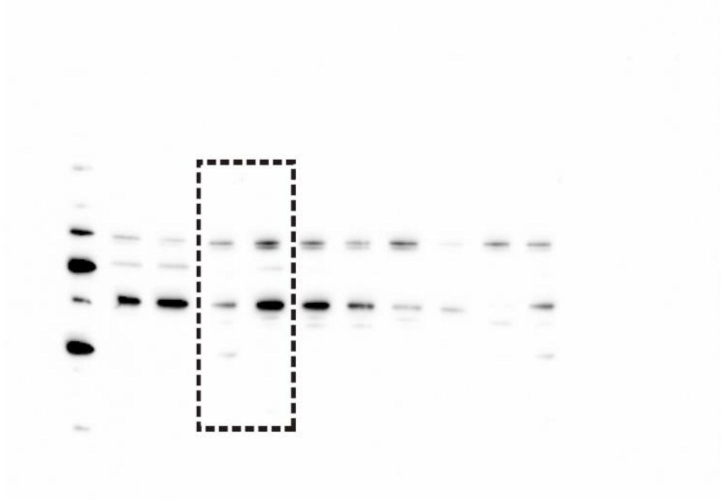

Figure 5D

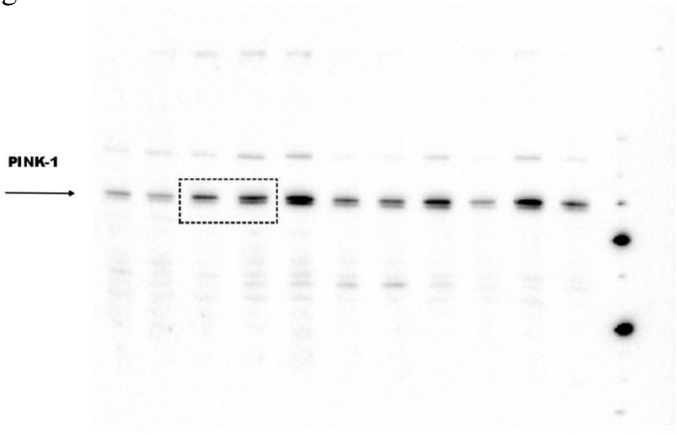

Supplement: Supplementary Figures [file srep44430-s1.pdf]
